# Local hyperexcitability of C-nociceptors may predict responsiveness to topical lidocaine in neuropathic pain

**DOI:** 10.1371/journal.pone.0271327

**Published:** 2022-07-14

**Authors:** Gunther Landmann, Lenka Stockinger, Benjamin Gerber, Justus Benrath, Martin Schmelz, Roman Rukwied

**Affiliations:** 1 Centre for Pain Medicine, Swiss Paraplegic Centre, Nottwil, Switzerland; 2 Department of Anesthesiology and Intensive Care, University Medical Centre Mannheim, University of Heidelberg, Heidelberg, Germany; 3 Department of Experimental Pain Research, Mannheim Center for Translational Neurosciences, Medical Faculty Mannheim, University of Heidelberg, Heidelberg, Germany; University of Würzburg, GERMANY

## Abstract

We explored whether increased C-nociceptor excitability predicts analgesic effects of topical lidocaine in 33 patients with mono- (n = 15) or poly-neuropathy (n = 18). Excitability of C-nociceptors was tested by transcutaneous electrical sinusoidal (4 Hz) and half sine wave (single 500 ms pulse) stimulation delivered to affected and non-affected sites. Analgesic effects of 24 hrs topical lidocaine were recorded. About 50% of patients reported increased pain from symptomatic skin upon continuous 4 Hz sinusoidal and about 25% upon 500 ms half sine wave stimulation. Electrically-evoked half sine wave pain correlated to their clinical pain level (r = 0.37, p < 0.05). Lidocaine-patches reduced spontaneous pain by >1-point NRS in 8 of 28 patients (p < 0.0001, ANOVA). Patients with increased pain to 2.5 sec sinusoidal stimulation at 0.2 and 0.4 mA intensity had significantly stronger analgesic effects of lidocaine and in reverse, patients with a pain reduction of >1 NRS had significantly higher pain ratings to continuous 1 min supra-threshold sinusoidal stimulation. In the assessed control skin areas of the patients, enhanced pain upon 1 min 4 Hz stimulation correlated to increased depression scores (HADS). Electrically assessed C-nociceptor excitability identified by slowly depolarizing electrical stimuli might reflect the source of neuropathic pain in some patients and can be useful for patient stratification to predict potential success of topical analgesics. Central neuronal circuitry assessment reflected by increased pain in control skin associated with higher HADS scores suggest central sensitization phenomena in a sub-population of neuropathic pain patients.

## Introduction

Neuropathic pain is a common complaint of patients with peripheral neuropathy and considered one of the most disabling neuropathic symptoms [1]. Neuropathic pain is caused by a lesion or disease of the somatosensory nervous system [2], but no correlation could be identified between pain intensity and severity of neuropathy [3, 4]. Functional tests evaluating the contribution of specific populations of primary afferent neurons to the neuropathy include quantitative sensory testing (QST) [5], pain-related evoked potentials (PREPs) for A-delta nociceptor functioning [6], laser doppler imaging (LDI) for C-nociceptor activation [7], and single nerve fiber recordings (microneurography) to identify spontaneous nociceptor discharges in neuropathic pain patients. These methods allow to determine the severity of neuropathy for different sensory pathways, but as of yet the mechanisms differentiating between painful and painless neuropathy are unclear [8, 9].

Cutaneous electrical stimulation is generally used to activate peripheral axons. The geometry of the electrodes and the temporal stimulation profile is critical for a selective activation of peripheral nerve fibers [10–14]. Slowly depolarizing transcutaneous currents of low-intensity with 4 Hz sinusoidal stimulation profile as well as single 500 ms half sine wave pulses can be used to stimulate nociceptors in human skin [15–18]. While single supra-threshold 500 ms half sine wave pulses induce a burst of action potentials, at 4 Hz sinusoidal stimulation single action potentials are evoked per sine wave [17]. Upon ongoing 4 Hz sinusoidal stimulation a profound adaptation of pain is observed in healthy subjects, whereas ratings in chronic pain or itch patient can increase [18, 19]. Here, we used these electrical stimuli to assess C-nociceptor excitability in patients suffering from neuropathic pain comparing affected and non-affected skin sites and correlate it to their acute pain level and to maxim pain in the last 7days.

Assuming that our excitability tests primarily assess the most superficially located skin nociceptors we hypothesized that topical lidocaine as a potent sodium-channel blocker widely used in the treatment of neuropathic pain [20] should be particular effective in those patients revealing hyperexcitable nociceptors. As the number to treat neuropathic pain is relatively high for topical lidocaine [21], it would be helpful to identify those patients with a higher chance of success. We therefore explored electrically evoked C-nociceptor activation profiles in neuropathic and non-affected skin of chronic neuropathic pain patients in correlation to pain relief upon topical lidocaine and to the patients’ level of spontaneous pain as well as psychological traits of depression and anxiety.

## Materials and methods

The Institutional Review Board of the North-West Ethics Committee (Switzerland) and the Ethics Committee II at the Medical Faculty Mannheim of the University of Heidelberg (Germany) approved the study protocol (project #2016–02048 and #2016-568N-MA). All institutional and governmental ethical regulations for human research were considered and the research conducted in accordance with the Declaration of the World Medical Association. Patients were informed about the study procedure and signed written informed consent.

### Patients

Subjects suffering from chronic pain were recruited at the Center for Pain Medicine in Nottwil (Switzerland) and the Pain Center of the Department of Anesthesiology at the Medical Faculty Mannheim of the University of Heidelberg (Germany). Inclusion criteria of the patients were (i) age 18 to 65 years, (ii) sufficient understanding of German language, and (iii) definite or at least probable peripheral neuropathic pain according the current criteria [22]. Exclusion criteria were (i) any underlying neurological or pain disorder other than stated in [22] and (ii) severe psychiatry disorder requiring inpatient treatment interfering with the study procedures.

### Clinical examination

All patients underwent a comprehensive neurological interview including pain history and neurological disease. Experienced physicians and neurologists (G.L. and B.G.) performed a clinical neurological examination, which included the assessment of muscle power, reflexes and sensory profiles (aesthesia and algesia). Briefly, mechanical and cold dysesthesia/allodynia, and pinprick hyperalgesia were identified using a standardized brush (Somedic, Horby, Sweden), a metal roll (length 3.5 cm, diameter 2.5 cm) kept at room temperature (20–23°C), and the tip of a Neuropen^®^ tester (Owen Mumford, Chipping Norton, United Kingdom). Diagnosis of peripheral neuropathic pain was made according the medical history, clinical examination and concomitant current criteria of neuropathic pain [22]. Patients were stratified into the major group “mono-neuropathy” (n = 15) and “poly-neuropathy” (n = 18), respectively, of which “mono-neuropathy” patients comprised also n = 5 patients with radiculopathy and the “poly-neuropathy” group comprised n = 2 patients with Guillain-Barré syndrome and n = 1 plexopathy patient (Table 1). In addition to the clinical examination, patients were requested to estimate on a numeric rating scale (NRS) with the endpoints 0 (no pain) and 10 (worst pain imaginable) the maximum pain they had perceived within the last 7 days.

**Table 1:** Clinical characteristics of the patients.

	Number (percent) of patients
Gender	
Women	15 (45%)
Men	18 (55%)
Age (years)	
Mean (± SD)	57 (± 12)
Pain duration (years)	
Mean (± SD)	6 (± 1.2)
Maximum pain (NRS, 0–10)	
last 7 days (mean ± SD)	5.6 ± 2.6
at visit (mean ± SD)	3.5 ± 2.5
Diagnosis	
Mononeuropathy	10 (30%)
Radiculopathy	5 (15%)
Polyneuropathy	15 (45%)
Plexopathy	1
Guillain-Barré	3
Pain medication	
No medication	5 (15%)
Anti-epileptics	13 (39%)
Opioids	16 (48%)
SNRI	10 (30%)
Tricyclics	6 (18%)
Other antidepressants	1
Analgesics	11 (33%)
Others (e.g. COX2)	2

### Questionnaires

A standardized pain history was obtained using the validated pain questionnaire and pain drawings of the German Society for the Study of Pain [23]. Anxiety and depression were assessed using the Hospital Anxiety and Depression Scale (HADS) with cutoff scores > 7 (anxiety) and > 5 (depression) [24, 25]. Health-related quality of life was determined by the SF-12 questionnaire with physical condition cutoff scores < 50 and mental health < 42 [26]. Chronic pain severity was assessed using the Graded Chronic Pain Scale (GCPS), including 5 hierarchical categories from grade 0 (no pain) to grade IV (high disability and severely limiting) [27], and the Mainz Pain Staging System [28], comprising a scoring system that includes pain characteristics, type of medication, previous consultation of physicians, pain-related intervention, hospital admission, and participation in rehabilitation programs, with which category scores of I to III were identified for pain chronicity in the patients [29, 30]. The medication of the patients was not changed during the study.

### Transcutaneous electrical nerve fiber stimulation

All patients underwent a training session to familiarize with the transcutaneous electrical stimulation paradigm and the use of the numeric rating scale (NRS, 0–10). In this session we delivered sinusoidal 4 Hz pulses for 2.5 sec at intensities of 0.05, 0.1 and 0.2 mA intensity, as well as single half sine wave pulses (500 ms duration) of 0.2, 0.4 and 0.8 mA intensity, respectively, to the patients non-affected forearm skin. Electrical stimuli were administered by means of a pair of bipolar platinum electrodes (diameter 0.4 mm, distance 2 mm, Nørresundby, Denmark) mounted in a 3D-printed applicator and held by the investigator attached to the patients’ skin surface. Sinusoidal 4 Hz and 500 ms half sine wave pulses were generated by a constant current stimulator (Digitimer DS5, Welwyn Garden City, UK) connected to a Digital-Analogue Converter (DAQ NI USB-6221, National Instruments, Austin, TX, USA) controlled by Dapsys 8 software (© Brian Turnquist, Bethel University, USA). For each stimulus paradigm patients rated the corresponding pain intensity on a numeric rating scale (NRS) with the endpoints 0 (no pain) and 10 (maximum pain that can be imagined). Data of the training session were not included in the analysis.

### Electrical stimulation protocol

Sinusoidal stimuli of 4 Hz were delivered to non-affected (control) and neuropathic skin for 2.5 sec (10 pulses) and with random intensities of 0.05–0.4 mA. Patients estimated the maximum perceived pain for each stimulus on the NRS (0–10). Continuous 4 Hz sinusoidal stimuli of supra-threshold intensity (definition see below) were delivered for 60 sec to non-affected (control) and painful (neuropathic) skin sites, respectively, and the corresponding pain intensity (NRS 0–10) recorded from the patient at 5 and 10 sec after stimulus onset, and thereafter in 10 sec intervals until stimulation termination. The current intensity for supra-threshold stimulation was defined by a pain of NRS 2–3 reported during the 10 sinusoidal pulses (2.5 sec, 4 Hz) assessed before from the patients’ painful (neuropathic) skin site. Thereafter, single half sine wave stimuli of 500 ms duration were administered with randomized current intensities of 0.2–0.4–0.6–0.8–1 mA to the non-affected and painful skin site, respectively.

All tests (sinusoidal and half sine wave stimulation) were performed twice for each current intensity and body site (average values calculated for analysis). In unilateral pain syndromes, such as mononeuropathy or radiculopathy, the contra-lateral body site was chosen as non-affected (control) skin site. In bilateral pain syndromes, such as polyneuropathy, a non-affected proximal limb area was chosen as control test area.

In addition to the recorded NRS data, delta values of perceived pain were calculated by subtraction between neuropathic and non-affected control skin sites for 2.5 sec sinusoidal 4 Hz pulses delivered with 0.2 and 0.4 mA, for the final 30 sec of continuous 1 min 4 Hz sinusoidal stimulation, and for half sine wave pulses of 0.8 and 1 mA intensity, respectively. Patients who perceived the stimuli more painful at the neuropathic site as compared to control skin by NRS differences ≥ 1 were classified “hyper-responsive”. Patients who perceived the stimuli less painful at the neuropathic sites (NRS differences ≤ -1) were grouped “hypo-responsive”, and patients who felt the stimuli similarly painful between the sites (NRS difference -1 to 1) were defined “normal-responsive”.

### Administration of a lidocaine patch

After assessment of transcutaneous electrical nerve fiber stimulation we applied a lidocaine 5% patch (Versatis^®^, Grünenthal, Stolberg (Germany) and Neurodol Tissugel^®^, IBSA Institut Biochimique SA, Pambio-Noranco (Switzerland)) on the patients’ neuropathic (painful) skin. Dependent on the area and field size of neuropathic pain, the patch covered 7x5 cm (finger/toes, n = 4), 7x10 cm (hand/foot dorsum, n = 19) or 14x10 cm (lower leg, n = 4), and the electrically tested site. Spontaneous (acute) pain (NRS 0–10) was recorded prior to patch application (t 0) and patients instructed to monitor the sensation at this site 30 min, 1h, 2h, 3h, 4h, 6h, 8h, 12h and 24h thereafter. Efficacy of the lidocaine 5% patch on spontaneous pain was assessed by calculating the difference between t 0 and the average pain estimates recorded during 3h and 12h lidocaine application, respectively. Thereupon, NRS differences ≤ -1 were defined as pain amelioration upon patch administration and NRS difference ≥ 1 as pain increase. NRS values in between indicated no effect on spontaneous pain.

### Statistical analysis

All data were analyzed using STATISTICA 7.1 software package (StatSoft Inc., Tulsa, US). Analysis of variance (ANOVA) was calculated between the factorial groups “neuropathic skin”–“stimulus intensity”–“stimulation time” and post hoc Fisher’s Least Significant Test (LSD) test. In addition, NRS of patients grouped into “hyper-responsive”–“hypo-responsive”–“normal-responsive” were analyzed by ANOVA and Fisher’s post-hoc comparison to identify significant differences between the groups (p < 0.05). The 95% confidence interval (CI) are mentioned in the text. Spearman’s rank correlation coefficient was assessed for NRS differences of electrically evoked pain between neuropathic and control skin site, maximum perceived pain within the last 7 days, acute (spontaneous) pain perceived at investigation time, and the pain relief upon lidocaine 5% patch. Also, sensory symptoms upon thermal and mechanical stimulation, as well as HADS and SF-12 questionnaire scores, respectively, were correlated to electrically and spontaneous (acute) pain. All values are depicted as mean ± SD.

## Results

### Patients, clinical examination and questionnaires

In total, 33 patients (n = 15 female and n = 18 male, average age 57 ± 12yrs) were recruited from both Pain Centers and subclassified into “mono-neuropathy” (n = 15) and “poly-neuropathy” (n = 18), respectively (Table 1). Patients suffered from chronic neuropathic pain for 6 ± 1.2yrs on average. At their visit, patients reported acute (spontaneous) pain of NRS 3.5 ± 2.5 (endpoints 0–10) and estimated their maximum pain perceived during the last 7 days with NRS 5.6 ± 2.6 on average.

Clinical characterization revealed at neuropathic skin sites dysesthesia to touch in n = 8 patients, allodynia to touch in n = 8 patients, and hypoesthesia to touch in 10 patients (Table 2). Hyperalgesia to pinprick stimuli was recorded in n = 19 patients. Cold hyperesthesia was reported from n = 5 patients and n = 19 patients revealed cold hypoesthesia.

**Table 2:** Clinical diagnostic, sensory symptoms, neuropathy profile, and test site of the patients.

Patient	Age	Sex	Pain duration (years)	Neuropathy	Detailed diagnosis	Painful site	Sensory symptoms on painful site	Sensory profile	Examinated painful site	Examinated painless site	Analgesia lidocaine patch
1	73	M	1	Polyneuropathy	Idiopathic PNP	Both lower legs and feet	Hypoaesthesia to cold Pinprick hyperalgesia	Thermal loss Mechanical gain	Dorsum foot L	Ventral forearm L	No
2	77	F	1	Polyneuropathy	Chemical induced PNP	Both feet and hands	Hypoaesthesia to cold Hypoaesthesia to touch Pinprick hyperalgesia	Thermal loss Mechanical loss Mechanical gain	Dorsum foot L	Ventral forearm L	Yes
3	57	F	n/a	Mononeuropathy	Lesion fibularis nerve	Foot R	Hyperaesthesia to cold Pinprick hyperalgesia	Thermal gain Mechanical gain	Dorsum foot R	Dorsum foot L	No
4	31	F	2	Mononeuropathy	Lesion fibularis nerve	Foot R	Hyperaesthesia to cold Pinprick hyperalgesia	Thermal gain Mechanical gain	Dorsum foot R	Dorsum foot L	Yes
5	65	F	1	Mononeuropathy	Lesion fibularis nerve	Lower leg and foot R	Hypoaesthesia to cold Hypoaesthesia to touch	Thermal loss Mechanical loss	Dorsum foot R	Dorsum foot L	n/a
6	80	M	n/a	Polyneuropathy	Idiopathic PNP	Both feet and hands	Hyperaesthesia to cold Hypoaesthesia to touch	Thermal gain Mechanical loss	Dorsum hand L	Ventral forearm L	No
7	52	M	n/a	Polyneuropathy	Idiopathic PNP	Both feet	n/a	n/a	Dorsum foot L	Ventral forearm L	n/a
8	55	F	3	Polyneuropathy	Chemical induced PNP	Both feet	Anaesthesia to temperature	Thermal loss	Dorsum foot L	Ventral forearm L	No
9	55	M	2.5	Polyneuropathy	Diabetic PNP	Both legs and feet	Hyperaesthesia to cold Hypoaesthesia to touch Pinprick hyperalgesia	Thermal gain Mechanical loss Mechanical gain	Ventral upper leg L	Ventral forearm L	No
10	62	F	2	Mononeuropathy	Lesion plantar nerve	Foot R	Allodynia to touch Pinprick hyperalgesia	Mechanical gain	Plantar foot R	Plantar foot L	Yes
11	77	F	4	Polyneuropathy	Chemical induced PNP	Both feet and hands	Allodynia to touch	Mechanical gain	Dorsum foot R	Upper arm R	No
12	70	F	n/a	Polyneuropathy	Chemical induced PNP	Both feet	Pinprick hyperalgesia	Mechanical gain	Dorsum foot R	Ventral forearm R	n/a
13	72	F	n/a	Polyneuropathy	Diabetic PNP	Both feet	Hypoaesthesia to cold	Thermal loss	Dorsum foot R	Ventral forearm R	n/a
14	49	M	22	Mononeuropathy	Lesion peroneal nerve	Lateral lower leg and dorsal foot L	Hypoaesthesia to cold Hypoaesthesia to touch Pinprick hypoalgesia	Thermal loss Mechanical loss	Dorsum foot L	Dorsum foot R	n/a
15	63	M	11	Polyneuropathy	Guillain-Barré syndrome	Both feed, hand R	Hypoaesthesia to cold Hypoaesthesia to touch Pinprick hypoalgesia	Thermal loss Mechanical loss	Dorsum foot L	Ventral upper leg L	No
16	51	M	4	Radiculopathy	Root L5	Both feet	Hypoaesthesia to cold Hypoaesthesia to touch Pinprick hypoalgesia	Thermal loss Mechanical loss	Dorsum foot L	Dorsal hand L	No
17	57	M	13	Polyneuropathy	Idiopathic SFN	Both feed	Hypoaesthesia to touch Pinprick hyperalgesia	Mechanical loss Mechanical gain	Dorsum foot L	Upper arm L	No
18	42	F	1.5	Polyneuropathy	Inflammatory SFN	Both legs	Hypoaesthesia to cold Hypoaesthesia to touch Pinprick hyperalgesia	Thermal loss Mechanical loss Mechanical gain	Dorsum foot R	Dorsal forearm R	No
19	38	F	3	Mononeuropathy	Lesion peroneal nerve	Lower leg and foot L	Hyperesthesia to cold Hypoaesthesia to touch Pinprick hyperalgesia	Thermal gain Mechanical loss Mechanical gain	Dorsum foot L	Ventral upper leg L	No
20	57	m	6	Polyneuropathy	Idiopathic PNP	1^st^ to 3^rd^ toes bilateral	Hypoaesthesia to cold Dysaesthesia to touch Pinprick hypoalgesia	Thermal loss Mechanical gain Mechanical loss	1^st^ toe R	Lower leg R	No
21	55	F	8	Polyneuropathy	Guillain-Barré syndrome	Both lower legs and feet	Anaesthesia to cold Dysaesthesia to touch Pinprick hypoalgesia	Thermal loss Mechanical gain Mechanical loss	Dorsum foot R	Ventral upper leg R	Yes
22	56	M	0.5	Polyneuropathy	Guillain-Barré syndrome	Foot L	Hypoaesthesia to cold Allodynia to touch Pinprick hypoalgesia	Thermal loss Mechanical gain Mechanical loss	Dorsum foot L	Shoulder L	Yes
23	64	M	0.5	Mononeuropathy	Lesion radial nerve	Radial nerve distribution hand R	Hypoaesthesia to cold Dysaesthesia to touch Pinprick hypoalgesia	Thermal loss Mechanical gain Mechanical loss	Radial nerve distribution hand R	Dorsal forearm L	Yes
24	63	M	2	Polyneuropathy	Diabetic SFN	All toes bilateral	Hypoaesthesia to cold Dysaesthesia to touch Pinprick hypoalgesia	Thermal loss Mechanical gain Mechanical loss	1st toe L	Upper arm L	No
25	53	F	4	Mononeuropathy	Lesion saphenous nerve	Saphenous nerve distribution R	Hypoaesthesia to cold Allodynia to touch Pinprick hyperalgesia	Thermal loss Mechanical gain	Medial lower leg R	Medial lower leg L	No
26	51	M	32	Plexopathy	arm plexus lesion lower part	Ulnar nerve distribution L	Anaesthesia to cold Allodynia to touch Pinprick hyperalgesia	Thermal loss Mechanical gain	4^th^ finger dorsal L	4^th^ finger dorsal L	No
27	63	F	6	Radiculopathy	Root L5	L5 dermatome lower leg L	Hypoaesthesia to cold Dysaesthesia to touch Pinprick hyperalgesia	Thermal loss Mechanical gain	Ventro-lateral lower leg L	Ventral upper leg L	No
28	61	F	12	Polyneuropathy	Idiopathic SFN	Both feet	Hypoaesthesia to cold Allodynia to touch Pinprick hyperalgesia	Thermal loss Mechanical gain	Dorsum foot R	Ventral upper leg R	Yes
29	61	M	4	Radiculopathy	Root L5	L5 dermatome R	Hypoaesthesia to cold Dysaesthesia to touch Pinprick hyperalgesia	Thermal loss Mechanical gain	Dorsum foot R	Dorsum foot L	No
30	42	M	0.5	Radiculopathy	Root L4	Ventro-medial lower leg R	Hypoaesthesia to cold Allodynia to touch Pinprick hyperalgesia	Thermal loss Mechanical gain	Ventro-medial lower leg R	Radial forearm R	No
31	23	M	2	Polyneuropathy	Idiopathic SFN	Both feet	Dysaesthesia to touch Pinprick hyperalgesia	Mechanical gain	Dorsum foot R	Ventral upper leg R	No
32	61	M	6	Mononeuropathy	Lesion ulnar nerve	Ulnar nerve distribution L	Allodynia to touch Pinprick hyperalgesia	Mechanical gain	Dorsal ulnar hand L	Dorsal ulnar hand R	Yes
33	48	M	5	Radiculopathy	Root L5	1st toe L	Dysaesthesia to touch Pinprick hyperalgesia	Mechanical gain	1st toe L	Ventral upper leg L	No

Patients’ etiology of neuropathy, sensory profile, test areas of affected (neuropathic/painful) and non-affected (control) skin sites, and presence of lidocaine 5% patch analgesia defined as reduction of acute (spontaneous) pain at visit by NRS values exceeding 1. Note that 5 patients did not return the questionnaire and in 1 patient the sensory profile was not assessed (n/a).

### Self-administered questionnaires

HADS scores of the patients indicated elevated levels of anxiety (9.4 ± 4.2) and depression (8.7 ± 4.4). SF-12 values indicated low physical (29.6 ± 8.6) and mental (43.7 ± 10.9) health related quality of life (Table 3). The vast majority of patients reported a high degree of pain severity (GCPS, 44% grade 4) and pain chronicity (MPSS, 65% score III).

**Table 3:** Questionnaire scores (HADS, SF-12) and pain scales (GCPS, MPSS) of the patients.

Assessment (number of patients)	Mean score ± SD (percent of patients)
HADS (n = 32)	
Anxiety	9.4±4.2
Depression	8.7±4.4
SF-12 (n = 30)	
PCS	29.7±8.9
MCS	43.8±11.3
VAS (CPGS, n = 20)	67.7±14.2
CPGS (n = 30)	
Grade 1	1 (3%)
Grade 2	7 (23%)
Grade 3	9 (30%)
Grade 4	13 (43%)
MPSS (n = 20)	
Score I	1 (5%)
Score II	6 (30%)
Score III	13 (65%)

VAS: Visual analog scale; HADS: hospital anxiety and depression scale; SF-12: short-form SF-12 health survey; PCS: physical component summary; MCS: mental component summary; CPGS: chronic pain grading scale; MPSS: Mainz pain staging system.

### Pain upon transcutaneous electrical stimulation

**Fig 1:**
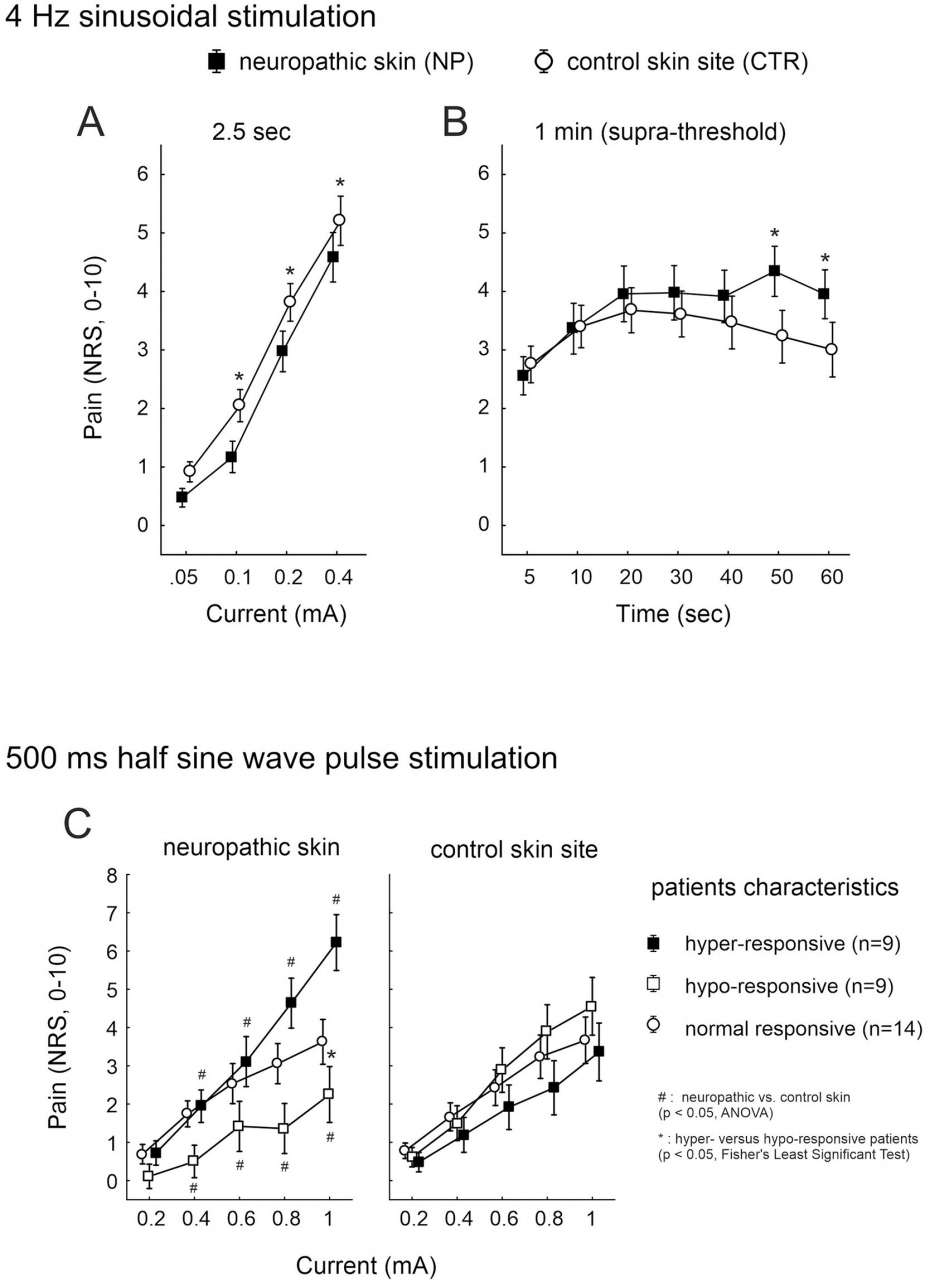
Pain to 4 Hz sinusoidal and 500 ms half sine wave stimulation. Electrically evoked pain (NRS, 0–10) recorded from the neuropathic (solid squares) and control (open circles) skin sites of 33 patients upon (A) 2.5 sec 4 Hz sinusoidal pulses of 0.05 to 0.4 mA, and (B) upon continuous (1 min) 4 Hz sinusoidal stimulation of supra-threshold intensity. (C) Pain recorded from neuropathic (left panel) and control skin (right panel) in response to 500 ms single half sine wave pulses of 0.2 to 1 mA intensity. Patients that reported increased pain at neuropathic compared to control skin sites upon stimulation (delta NRS > 1) were grouped “hyper-responsive” (solid squares), patients with less sensitivity (delta NRS < -1) “hypo-responsive” (open squares), and patients without NRS differences between the sites (delta NRS < 1and > -1) classified “normal” (open circles). Hash symbols indicate significant differences between neuropathic and control skin (p < 0.05, ANOVA), asterisks indicate significant post-hoc comparisons between the patients’ groups (p < 0.05, Fisher’s Least Significant Test).

Sinusoidal stimuli of 4 Hz and 2.5 sec (10 pulses) induced current intensity dependent pain (p < 0.0001, CI 0.293, ANOVA) and a significant difference between the patients’ neuropathic *versus* control skin site (p = 0.05, CI 0.41, ANOVA). Pain estimates were significantly lower in neuropathic skin compared to the non-affected sites at current intensities of 0.1 to 0.4 mA (p < 0.01, Fisher’s Least Significant Test, CI 0.48 and 0.46, Fig 1A).

Dependent on the NRS during 2.5 sec of 4 Hz stimuli and as defined above, we administered supra-threshold 4 Hz sinusoidal pulses continuously for 1 min to the patients’ skin sites. A current intensity of 0.1 mA was applied in n = 6 patients, 0.2 mA in n = 22, and 0.4 mA in n = 6 patients. Notably, in the latter group (0.4 mA stimulation), n = 3 patients reported an NRS < 1 upon the 10 sinusoidal pulses delivered at their painful skin site.

During continuous supra-threshold 4 Hz sinusoidal stimulation, pain recorded from neuropathic and control skin was significantly different over time (p < 0.02, ANOVA, CI 0.32 and 0.29). In particular, pain recorded from control skin declined after 20 sec of stimulation, but remained elevated in neuropathic skin (Fig 1B), revealing significance at 50 and 60 sec stimulation (p < 0.002, Fisher’s LSD test, CI 0.58 and 0.63). Mean pain ratings in neuropathic and control skin for the half sine wave pulses did not differ significantly, however, this was based on local sensitization in some and desensitization in other patients, cancelling out each other. Half sine evoked pain in neuropathic skin exceeded the control in 9 patients (“hyper-responsive” group: average NRS 5 in neuropathic versus NRS 3 in control skin, p < 0.0001, ANOVA, 0.69 and 0.45, Fig 1C). In another 9 patients the opposite was true (“hypo-responsive”) and for 14 patients no difference between the two skin sites was found (“normal”). Calculation of the slope of current intensity dependent pain increase (subtraction NRS 0.2 mA from NRS 1 mA) indicated a significantly steeper incline of pain in neuropathic skin of “hyper-responsive” patients compared to “hypo- and normal-responsive” patients, respectively (p < 0.001, ANOVA, CI 0.83 and 0.77).

**Fig 2:**
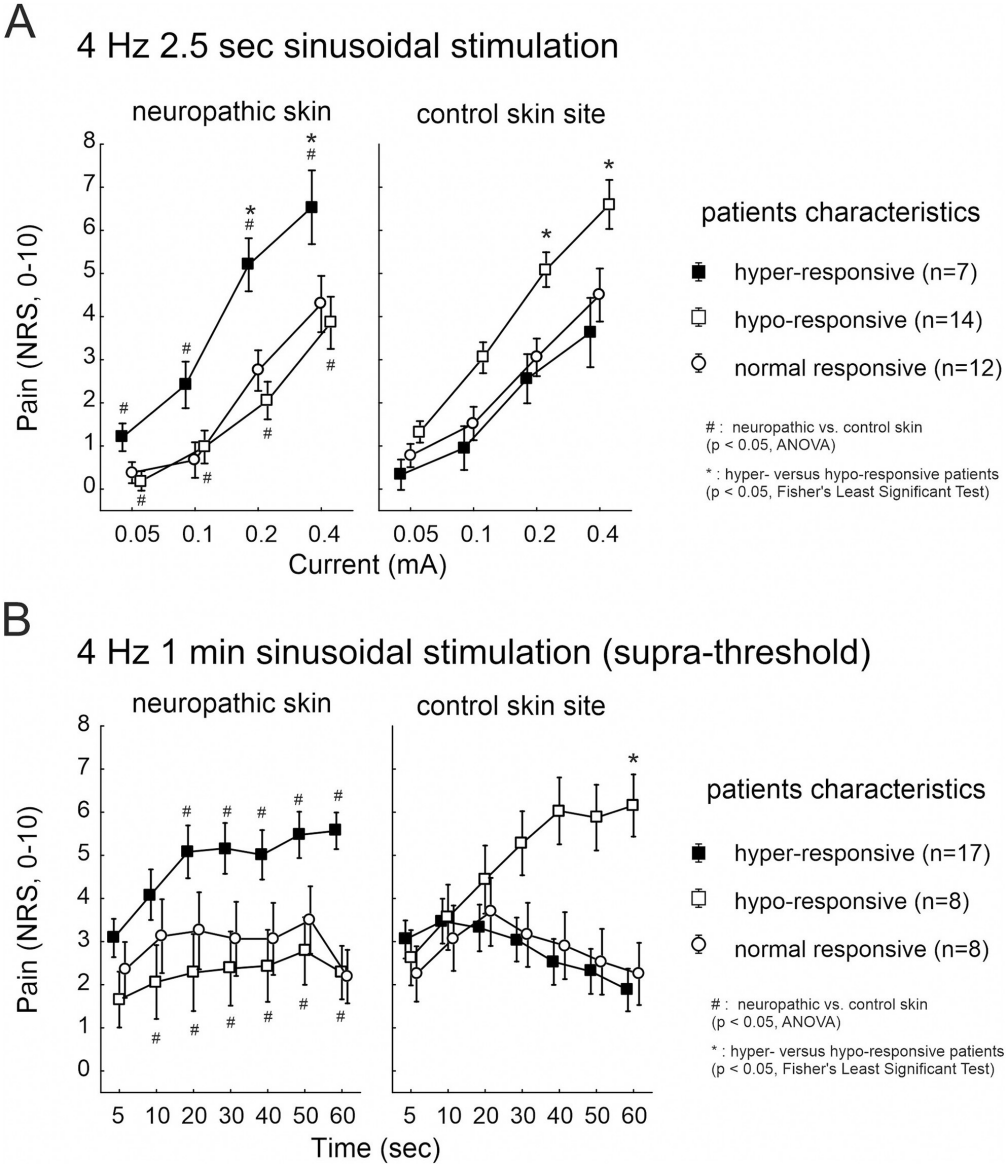
Pain upon 4 Hz sinusoidal pulses. Electrically evoked pain (NRS, 0–10) recorded from the neuropathic (left panel) and control (right panel) skin sites upon (A) 2.5 sec 4 Hz sinusoidal pulses of 0.05 to 0.4 mA (n = 33), and (B) continuous 1 min 4 Hz sinusoidal pulses of supra-threshold intensity (n = 33). Patients that reported increased pain at neuropathic compared to control skin sites upon stimulation (delta NRS > 1) were grouped “hyper-responsive” (solid squares), patients with less sensitivity (delta NRS < -1) “hypo-responsive” (open squares), and patients without NRS differences between the sites (delta NRS < 1and > -1) classified “normal” (open circles). Hash symbols indicate significant differences between neuropathic and control skin (p < 0.05, ANOVA), asterisks indicate significant post-hoc comparisons between the patients’ groups (p < 0.05, Fisher’s Least Significant Test).

NRS values calculated between neuropathic and control skin indicated a significant interaction between the factorial groups “hyper-responsive patients” (definition see above, delta NRS ≥ 1), the delivered “current intensity” and the investigated “skin site” (p < 0.0001, ANOVA) for pain upon 2.5 sec 4 Hz sinusoidal pulses. Average pain in “hyper-responsive” patients (n = 7) was NRS 5–6 in neuropathic *versus* NRS 2–3 in control skin (p < 0.05, Fisher’s LSD test, CI 1.52 and 0.86). In contrast, patients classified “hypo-responsive” (n = 14, delta NRS ≥ -1) reported NRS 2–4 in neuropathic compared to NRS 5–6 in control skin (p < 0.05, Fisher’s LSD test, CI 0.38 and 0.45, Fig 2A).

Continuous 1 min 4 Hz supra-threshold sinusoidal stimulation evoked in 17 patients enhanced pain, particularly during 30 to 60 sec of stimulation, in neuropathic (average NRS 5, “hyper-responsive” group) compared to control skin sites (average NRS 2, p < 0.0001, CI 0.53 and 0.48, ANOVA). 8 patients reported that the 4 Hz stimuli were similarly painful in neuropathic and control skin (“normal-responsive”, average NRS 2–3), whereas 8 patients, however, indicated stronger pain at the control sites (average NRS 5–6, p < 0.0001, Fisher’s LSD test, CI 0.77 and 0.53, Fig 2B). We evaluated whether pain sensation upon sinusoidal stimulation was different between mono-neuropathy (MNP, n = 15) and poly-neuropathy (PNP, n = 18) patients. No significant NRS differences were calculated between the neuropathy-groups at the control skin sites (n.s., ANOVA). Similarly, NRS recorded from neuropathic skin sites was not significantly different between MNP and PNP patients (n.s., ANOVA).

### Relief of spontaneous pain upon a lidocaine 5% patch

**Fig 3:**
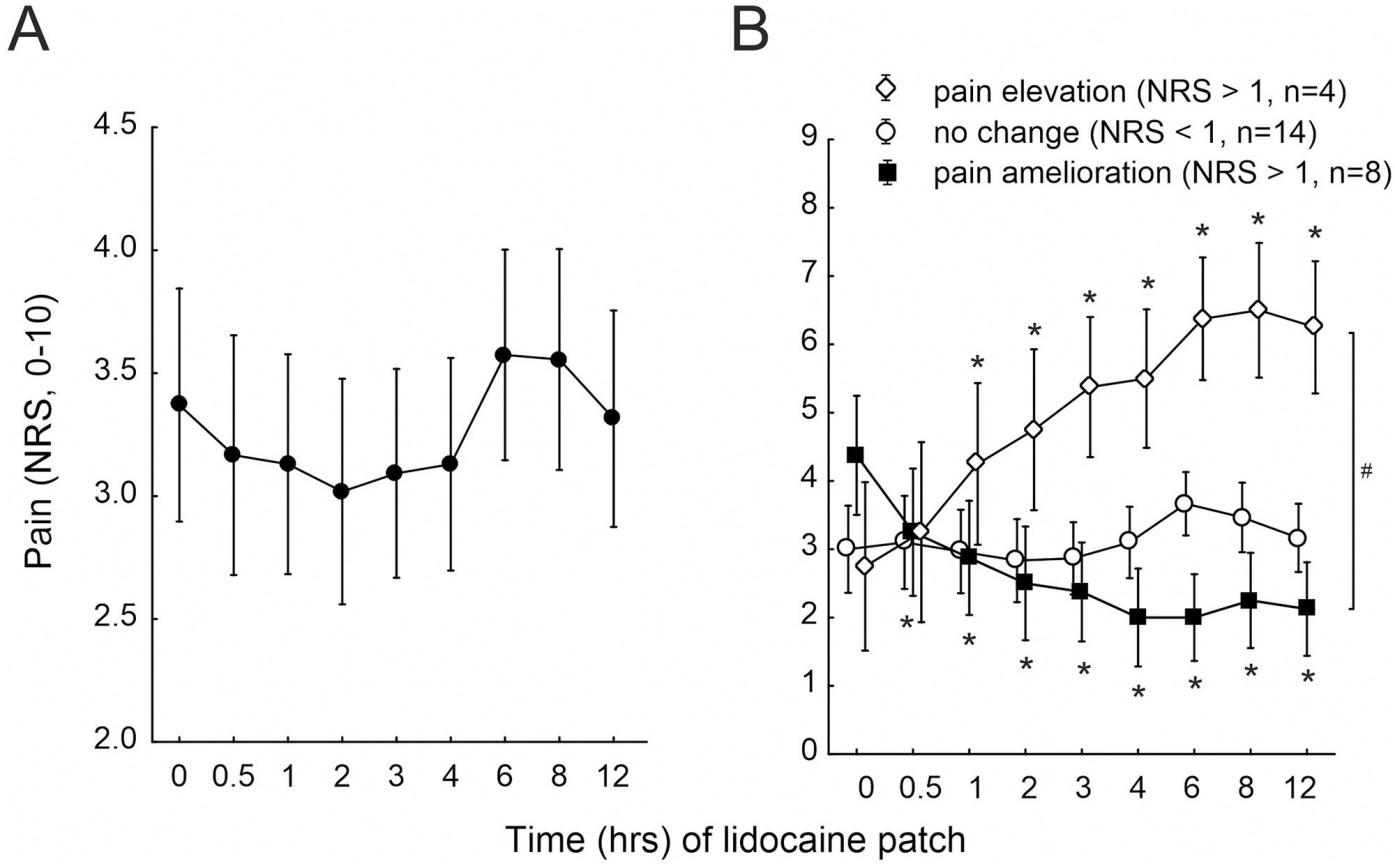
Pain in response to topical lidocaine. (A) Evaluation of acute (spontaneous) pain (NRS, 0–10) of 27 patients at the time of visit (t = 0) and upon a lidocaine 5% patch applied to the assessed neuropathic skin site at 0.5–12 hours after application. (B) Acute (spontaneous) pain (NRS, 0–10) at the time of investigation (t 0) and in response to a lidocaine 5% patch at 0.5–12 hours after application in patients that reported a pain reduction exceeding NRS 1 compared to t 0 (“pain amelioration”, solid squares), pain enhancement of t 0 exceeding NRS 1 (“pain elevation”, open diamonds), and patients without lidocaine effect on t 0 (NRS changes– 1 to 1, open circles). Hash symbol indicates significant differences between the patients’ group (p < 0.0001, ANOVA) and asterisks indicate significance compared to t 0 acute (spontaneous) pain (p < 0.05, Fisher’s Least Significant).

Following pain assessment upon transcutaneous electrical stimulation, 27 patients received a lidocaine 5% patch attached to their neuropathic skin site. Prior to patch administration, acute (spontaneous) pain was rated by the patients on average NRS 3.4 ± 0.5 at the neuropathic skin site (t 0, Fig 3A). During 3 hours lidocaine 5%, patients reported a pain of NRS 3 ± 0.5 on average (n.s., ANOVA). We calculated a potential pain reduction upon lidocaine for each patient by subtracting the average NRS scores recorded within 3 hours patch administration from acute (spontaneous) pain. A reduction by a value exceeding NRS 1 was reported from n = 8 patients (“pain amelioration”), 15 patients did not report a change, whereas 4 patients recorded an increase of pain by NRS > 1 during patch administration (“pain elevation”). Notably, 2 patients of the latter group had no pain at the time of lidocaine 5% patch administration (t 0). A significant interaction could be analyzed for the groups of patients between the time of lidocaine administration and the reduction of acute (spontaneous) pain (p < 0.0001, CI 0.49, ANOVA, Fig 3B and Table 2).

**Fig 4:**
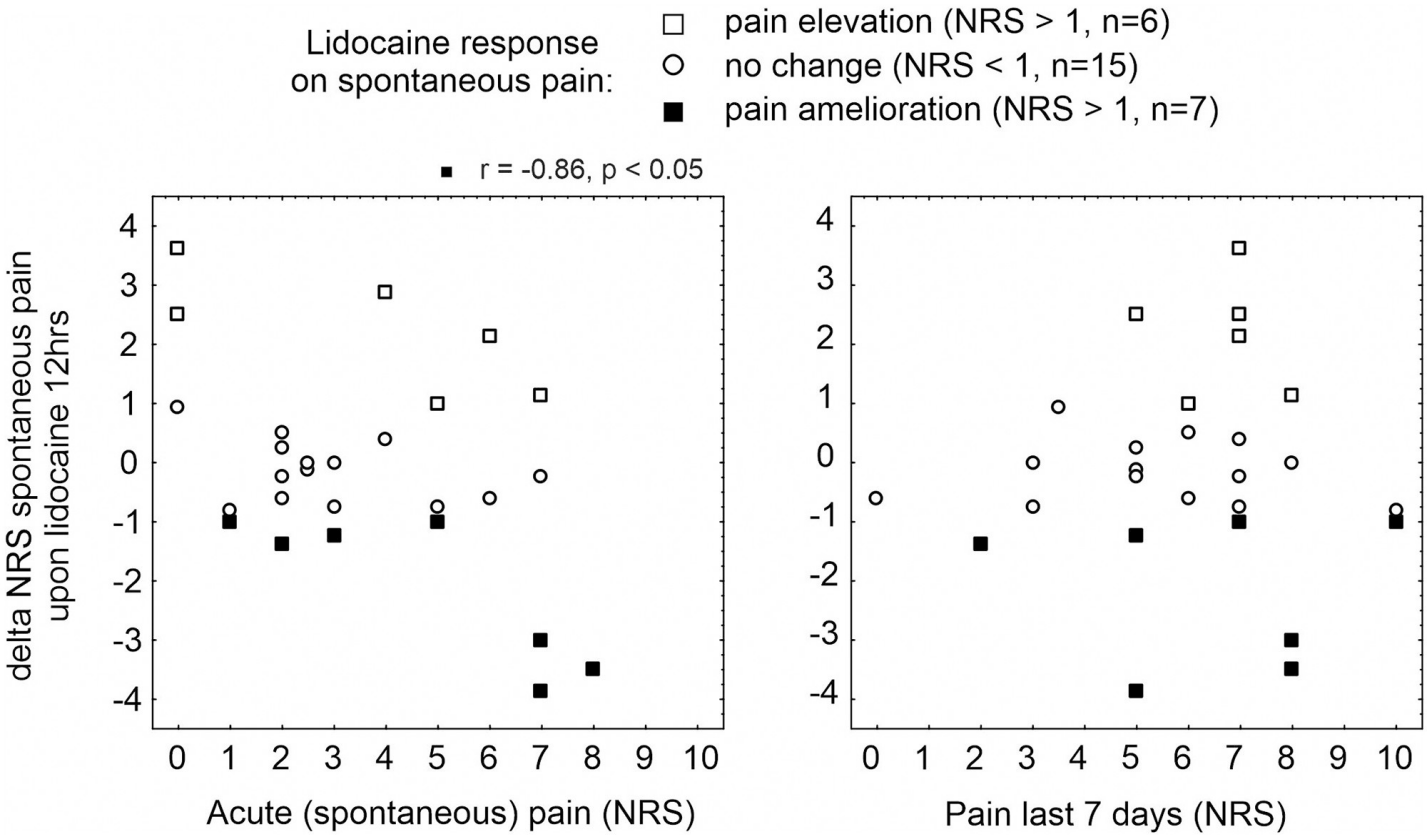
Data correlation analysis. Spearmans rank correlation between pain reduction upon 12 hours lidocaine 5% patch (delta NRS, -10 to 10) and magnitude of acute (spontaneous) pain (left panel) or maximum pain perceived during 7 days (right panel), respectively, in patients reporting a pain reduction (“pain amelioration”, solid squares, p < 0.05), pain facilitation (“pain elevation”, open squares) or no alteration of acute pain (“no change”, open circles). Note that 3 patients who reported about a pronounced beneficial lidocaine effect also had high scores of acute (spontaneous) and maximum 7-days pain.

We evaluated whether the pain reduction upon lidocaine was associated with the magnitude of patients’ acute (spontaneous) or maximum pain perceived during the last 7 days prior assessment, respectively. NRS changes evoked by 12 hours lidocaine patches correlated significantly to spontaneous pain intensity in 7 patients who reported a pain amelioration (Spearmans rank correlation r = -0.9, p < 0.02, Fig 4). Of note, pain relief was particularly strong in 3 patients (2 MNP and 1 PNP) who also recorded strong pain (NRS 5 and NRS 8) within the last 7 days (Spearmans rank correlation, n.s., Fig 4).

**Fig 5:**
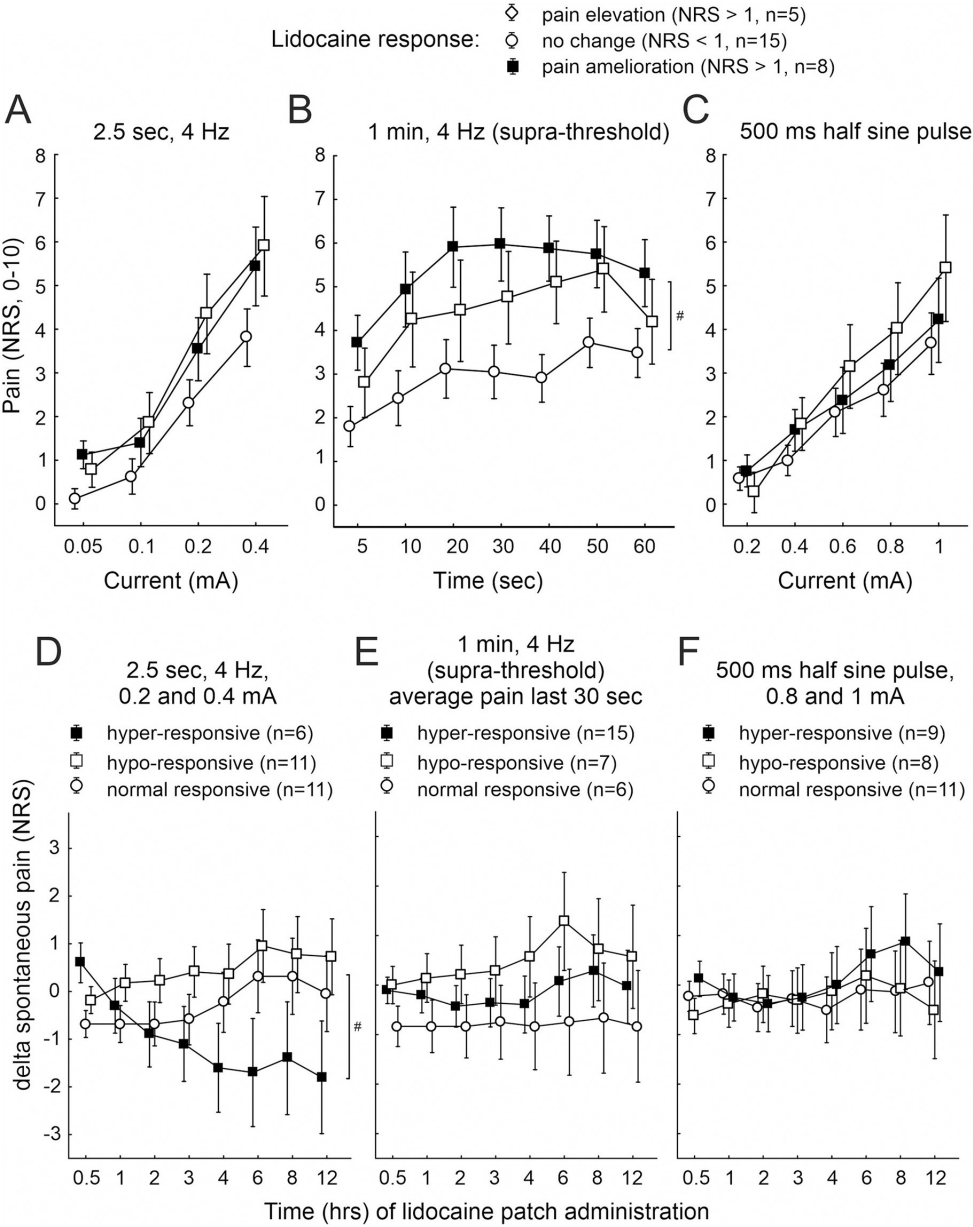
Comparison of electrically induced pain in lidocaine responding patients and lidocaine effects in patients sensitized to electrical stimulation. Electrically evoked pain (NRS, 0–10) recorded from neuropathic skin sites upon (A) 2.5 sec 4 Hz sinusoidal pulses of 0.05 to 0.4 mA, (B) 1 min 4 Hz sinusoidal pulses of supra-threshold intensity, and (C) 500 ms single half sine wave pulses of 0.2 to 1 mA in patients who reported about a reduction of acute (spontaneous) pain during 3 hours lidocaine patches (“pain amelioration”, solid squares), pain facilitation (“pain elevation”, open squares) or no alteration of acute pain (“no change”, open circles). Sinusoidal 1 min stimulation evoked significantly more pain in patients revealing a lidocaine effect (“pain amelioration”) than in patients without a response (p < 0.03, ANOVA, marked by hash symbol). Alteration of acute (spontaneous) pain due to a lidocaine 5% patch (delta NRS, -10 to 10) during 0.5–12 hours of application depicted in patients with “hyper-responsive” (solid squares”), “hypo-responsive” (open squares) or “normal” (open circles) neuropathic skin sites to (D) 2.5 sec 4 Hz sinusoidal pulses of 0.2 and 0.4 mA, (E) within the last 30 sec of 1 min 4 Hz supra-threshold sinusoidal pulses, and (F) 500 ms single half sine wave pulses of 0.8 and 1 mA. Hash symbol indicates a significant difference of acute (spontaneous) pain reduction by the lidocaine patch between “hyper-responsive” and “hypo-” or “normal-responsive” patients (p < 0.005, ANOVA).

Of our further interest was to identify, on one hand, whether pain NRS upon transcutaneous electrical stimulation of the neuropathic skin site was different in patients who reported a lidocaine pain relief within 3 hours lidocaine patches (Fig 5A–5C), and on the other hand to evaluate whether patients being “hyper-responsive” to transcutaneous electrical stimulation at neuropathic skin sites revealed a particular susceptibility for a lidocaine patch pain relief (Fig 5D–5F).

Pain recorded during 2.5 sec sinusoidal and 500 ms half sine wave stimulation was not different between the groups of lidocaine responders (n.s., ANOVA, Fig 5A and 5C). Significantly elevated pain during 1 min supra-threshold sinusoidal stimulation was recorded from neuropathic skin in patients with a pain amelioration upon lidocaine (average maximum NRS 6 ± 1, n = 8) compared to patients without lidocaine effect (average maximum NRS 3 ± 0.6, n = 15, p < 0.03, CI 0.65 and 0.35, ANOVA, Fig 5B). Those patients who revealed a “hyper-sensitivity” to 2.5 sec sinusoidal stimuli of 0.2 and 0.4 mA intensity, on the other hand, responded with a significant pain amelioration during the lidocaine patch (average pain reduction by about NRS 2) compared to “hypo-” or “normal-responsive” patients (average pain reduction NRS 0, p < 0.005, CI 0.69 and 0.3, ANOVA, Fig 5D). No significant lidocaine effect on spontaneous pain was observed in patients “hyper-sensitive” during the final 30 sec of 1 min sinusoidal stimulation or 500 ms half sine wave pulses of 0.8 and 1 mA (n.s., ANOVA, Fig 5E and 5F).

No significant correlation was identified for pain relief during 3 hours lidocaine patch application and pain responses recorded between neuropathic and control skin sites during transcutaneous electrical stimulation, but patients being hyper-sensitive to electrical stimulation, particularly those with strong responses to 1 min sinusoidal pulses, showed trends of a lidocaine induced pain amelioration (Spearmans rank correlation r = -0.46, p = 0.08, S1 Fig). We finally analyzed whether increased pain sensitivities to electrical stimulation of neuropathic skin sites correlated with acute (spontaneous) or maximum 7-day pain, respectively. No significant correlations were found for 2.5 sec and 1 min 4 Hz sinusoidal stimulation, even though patients who responded to lidocaine patches with pain amelioration revealed a Spearmans rank correlation of r = 0.69 (p = 0.05) between 2.5 sec sinusoidal and acute (spontaneous) pain (S2A Fig). Notably, a significant correlation was found in patients with neuropathic skin hyper-sensitivity to 500 ms half sine wave stimulation and the maximum 7 days pain recording (Spearmans rank correlation r = 0.37, p < 0.05, S2C Fig).

### Data correlation with clinical examination profile and questionnaires

Sensory tests of cold hyperaesthesia, pinprick hyperalgesia and allodynia to touch of patients’ neuropathic skin sites were correlated to acute (spontaneous) and maximum 7-day pain, to pain amelioration upon 3 respectively 12 hours lidocaine 5% patches, and to pain evoked by transcutaneous electrical stimuli delivered at high current intensities (S1A Table). Electrically evoked pain did not correlate with any sensory profile. Acute (spontaneous) pain and pain relief upon 12 hours lidocaine correlated significantly with the presence of allodynia to touch (Spearmans rank correlation, p < 0.05). We also analyzed whether HADS depression and anxiety or SF-12 physical and mental questionnaire scores correlated to acute (spontaneous), maximum 7-days or electrically evoked pain (S1B Table). HADS depression and anxiety as well as SF-12 mental score correlated significantly with electrical pain from patients control skin sites during 20 sec of ongoing sinusoidal stimulation (Spearmans rank correlation, p < 0.05). In particular, patients with “hypo-responsive” neuropathic skin to 1 min supra-threshold sinusoidal stimuli revealed a positive correlation between HADS depression and electrically evoked pain of their control skin site during 20 sec sinusoidal stimulation (r = 0.83, p < 0.03). HADS anxiety scores were not significant in this group (n.s.), whereas “hyper-responsive” patients in contrast showed a significant correlation between HADS anxiety and sinusoidal control skin stimulation (r = 0.68, p < 0.01). Neither acute (spontaneous) nor maximum 7-days pain correlated significantly to the HADS and SF-12 questionnaire scores in any of the patients (S1B Table).

## Discussion

C-nociceptor excitability in neuropathic pain patients as assessed by slowly depolarizing electrical pulses was increased in symptomatic skin in about 1/3 of the patients. Patients that were hyper-responsive to short bursts of 4 Hz stimulation showed a significantly higher analgesic effect to topical lidocaine. Patients who reported a pain reduction of > 1 NRS to topical lidocaine had significantly higher pain ratings during ongoing 1 min 4 Hz sinusoidal stimulation, suggesting C-nociceptor excitability tested by slow electrical depolarization might be related to mechanisms generating neuropathic pain and could potentially improve patient selection for topical analgesic therapy.

### Sensitization profile upon transcutaneous electrical stimulation

Slowly depolarizing transcutaneous electrical stimulation (4 Hz sinusoidal profile and 500 ms half sine wave pulses) induced burning pain in healthy subjects, activated primary afferent C-nociceptors, and revealed substantial accommodation upon continuous 4 Hz sinusoidal stimulation [15, 17, 18]. We found in our patients that about 50% reported enhanced pain upon 4 Hz sinusoidal stimulation of their stimulated neuropathic skin sites. Half sine wave pulses delivered to neuropathic sites were perceived stronger in about 25% of patients, of which only half had also stronger pain during sinusoidal stimulation. Notably, ongoing 4 Hz sinusoidal stimuli were perceived increasingly intense, a phenomenon confirming our previous observation in chronic pain [18] but also chronic inflammatory pruritus patients, the latter reported strong itch upon electrical stimulation [19]. An enhanced axonal excitability in neuropathic skin of patients might be suggested, considering that signal transduction processes are circumvented by the electrical stimulation paradigm. It is difficult to precisely conclude which C-nociceptor sub-types are primarily affected in neuropathic skin sites, as both “polymodal” and “silent” nociceptors can be activated by sinusoidal stimuli whereas “polymodal” but not “silent” nociceptors respond to half sine wave pulses [17, 18]. However, potentially augmented peripheral excitatory input of nociceptors might not be limited to the symptomatic skin area (Fig 2B), also descending inhibitory circuits and central sensitization processes have to be considered contributing to electrically evoked and acute (spontaneous) pain in patients. We therefore implicated the analyses of questionnaires and clinical symptoms, respectively, and found that maximum 7-days pain reported by the patients correlated significantly with the presence of allodynia to touch, one clinically assessed cardinal symptom of central sensitization. Moreover, patients with a reduced sensitivity of neuropathic skin to continuous sinusoidal stimulation reported significantly enhanced pain evoked at their non-affected control skin sites. In these patients, pain recorded during 30 sec sinusoidal stimulation from this site positively correlated with the patients HADS-depression score, which might indicate a central rather peripheral mechanism of sensitization. The high prevalence of pain chronicity according to MPSS and the symptoms of anxiety and depression assessed by HADS were comparable to those reported in chronic non-cancer pain patients [31, 32]. Self-assessed pain related limitations in our cohort were in accordance to patients with uncontrolled neuropathic pain, of which 53% reported depression and 43% anxiety severely interfering their daily activities [33], collaborating the aspect of a central sensitization process.

No significant correlation was identified between electrically evoked pain to sinusoidal stimulation and the patients’ intensity of acute (spontaneous) or maximum 7-days pain, respectively. In contrast, a sub-population of patients reported significantly stronger pain upon a single 500 ms half sine wave pulse in neuropathic skin that positively correlated to their maximum 7-days pain. It could be hypothesized that significantly elevated pain at this body site upon a single electrical half sine wave stimulus indicated increased supra-threshold responses of mechanical- and heat-sensitive (“polymodal”) nociceptors being activated by this stimulation paradigm [17]. Enhanced supra-threshold excitability is not necessarily mirrored by elevated acute (spontaneous) pain, but perhaps influenced in the retrospect maximum 7-days pain. Notably, electrically evoked pain did not correlate to pinprick-, touch- or cold-sensitization. These sensory stimuli addressed specific nerve fiber classes that have only little contribution to half sine wave evoked pain [17], i.e. thinly myelinated A-delta fibers assessed by pinprick and cold, A-beta and low-threshold mechano-sensitive (LTM) C-fibers recruited by touch, and therefore magnitude of sensation upon supra-threshold electrical nociceptor activation may not correlate with perception intensity upon threshold activation of primary sensory afferents.

We were not able to provide a clear correlation between our electrically induced read-outs and the patients’ pain reported from neuropathic skin. This observation highlights the yet unsolved problem to mechanistically differentiate neuropathic patients with and without chronic pain [34]. However, a phenotyping of neuropathic pain in patients still may be considered based on our present results: i) none or only minor peripheral C-nociceptor pathology involvement (no alteration of electrically-induced pain), ii) pathologic C-nociceptor involvement (enhanced electrically-induced pain), iii) axonal sensitization (peripheral) of C-nociceptors (increasing pain upon ongoing sinusoidal stimulation), and iv) contribution of central sensitization (increased pain upon ongoing sinusoidal stimulation of non-neuropathic skin). The electrical stimulation paradigm thus would allow to characterize the patients as “hyper-responsive” (gain of C-nociceptor function), “hypo-responsive” (loss of C-nociceptor function), and “normal-responsive” (no alteration of C-nociceptor function).

### Analgesia by topical lidocaine

We further explored the efficacy of a topical lidocaine 5% patch on spontaneous (acute) and maximum 7-days pain and its correlation to electrically induced pain. There is a controversial discussion about the efficacy of topical lidocaine for the treatment of neuropathic pain (for review see [21]). Here, about 25% patients reported a mild amelioration of pain when defined as 1-point of 10 reduction in pain intensity. Pain reduction correlated to the magnitude of acute (spontaneous) pain but not to maximum pain perceived within 7 days. No correlation, however, was identified between lidocaine pain reduction and sensitivity of neuropathic skin sites to electrical stimulation, but the lidocaine effect on acute (spontaneous) pain revealed to be stronger in those patients who reported a 2.5 sec sinusoidal stimulation sensitization. This trend was not confirmed in patients being hyper-sensitive to 1 min ongoing sinusoidal stimuli, which could be related to an additional central pain processing involved in ongoing sinusoidal pain perception compared to the primarily peripheral mechanism most likely involved in 2.5 sec stimulation, which would apply also for 500 ms half sine wave pulses. However, no difference of lidocaine efficacy was observed in this group. Another explanation for differential correlation between lidocaine and electrical sensitization could be due to a variation of lidocaine-affinity to neurons displaying “resurgent” currents [35], which is the occurrence of persistent firing even in presence of clinically relevant high concentrations of exogenous use-dependent blockers [36]. Augmented resurgent currents were considered to participate in neuronal hyper-excitability and pain facilitation under pathologic conditions [37, 38], and thus possibly contributed also to elevated pain recorded from few of our patients during lidocaine patch administration.

On the other hand, patients reporting a beneficial effect of lidocaine experienced stronger pain in neuropathic skin upon 1 min sinusoidal but not half sine wave stimulation. Activation of C-nociceptors by 4 Hz sinusoidal pulses also provoked an axon-reflex mediated vasodilation [18], thus their distribution in the skin should be in close vicinity to arterioles located in deeper layers. A saturation of these zones with lidocaine by 12 hrs skin patch administration could be expected. The link between skin sensitization to sinusoidal stimulation and analgesia by topical lidocaine, therefore, indicated the potential role of nociceptors prone to sinusoidal stimulation (and consecutive vasodilation) involved in acute (spontaneous) neuropathic pain.

### Limitation

Slowly depolarizing ramp currents evoke a strong sodium channel NaV1.7 ramp current [39, 40] and thereby can activate unmyelinated C-fibers, as shown also by other labs [12, 14]. Co-activation of (thinly) myelinated peripheral nerves by this stimulation paradigm appears less likely due to the inactivation kinetics of NaV1.6 in those fibers. However, not all of our chronic neuropathic pain patients responded in one and the same fashion to slowly depolarizing electrical stimulation and topical lidocaine analgesia. Moreover, it remains unclear why only short lasting but not tonic 1 minute 4 H stimulation correlated to pain reduction. This may indicate the involvement of other, yet to be identified variables contributing to the pathophysiology of neuropathic pain and our observations made herein. The complexity and multifaceted factors of this disease are difficult to assess in a single experimental approach and make interpretation of the findings or potential therapeutic intervention difficult. Accordingly, an increase in the number of subjects may be needed to get a clearer picture and interpretation of the results, which would help to clarify those aspects that do not support the hypothesis.

## Conclusion

Our results suggest that hyperexcitability of C-nociceptors might contribute to clinical neuropathic pain in a subgroup of our patients. Analgesic effects of topical lidocaine were higher in patients positive for touch evoked allodynia, hyperexcitable to sine wave stimulation, and with higher baseline pain levels. These aspects might therefore be considered useful for a patient stratification. On the other hand, the link between higher anxiety and depression levels correlating to increasing pain ratings to 4 Hz sinusoidal stimulation in control skin highlights the important role of central nervous system circuitry in chronic neuropathic pain.

## Supporting information

S1 Fig Spearmans rank correlation. Analysis between pain reduction upon 3 hours lidocaine 5% patch (delta NRS, -10 to 10) and pain recorded from neuropathic (delta NRS from control skin sites) during (A) 2.5 sec 4 Hz sinusoidal pulses of 0.2 and 0.4 mA, (B) within the last 30 sec of 1 min 4 Hz supra-threshold sinusoidal pulses, and (C) upon 500 ms single half sine wave pulses of 0.8 and 1 mA. Patients are grouped “hyper-responsive” (solid squares”), “hypo-responsive” (open squares) or “normal responsive” (open circles) to transcutaneous electrical stimuli.
(TIF)

S2 Fig Spearmans rank correlation. Analysis between pain from neuropathic skin sites (delta NRS neuropathy vs. control) and acute (spontaneous) pain (left panel) or maximum pain perceived during 7 days (right panel) upon (A) 2.5 sec 4 Hz sinusoidal pulses of 0.2 and 0.4 mA, (B) within the last 30 sec of 1 min 4 Hz supra-threshold sinusoidal pulses, and (C) upon 500 ms single half sine wave pulses of 0.8 and 1 mA. A significant correlation was identified between neuropathic skin site pain upon half sine wave stimulation and the 7-day maximum pain (p < 0.05). Scatterplot depicts patients’ groups by their lidocaine-patch response of acute (spontaneous) “pain-amelioration” (solid squares), “pain elevation” (open squares) or “no change” (open circles), respectively.
(TIF)

S1 Table Data correlation. (A) Spearmans rank correlation between sensory profiles and NRS scores. (B) Spearmans rank correlation between questionnaire scores (HADS and SF-12) and acute (spontaneous) pain, 7-day pain, and electrically evoked pain in neuropathic (NP) and control (ctr) skin.
(DOCX)
