# Supplementary figures and images for: Local hyperexcitability of C-nociceptors may predict responsiveness to topical lidocaine in neuropathic pain
Source: PLoS One. 2022 Jul 14;17(7):e0271327. doi: 10.1371/journal.pone.0271327 (PMC9282664; doi:10.1371/journal.pone.0271327)

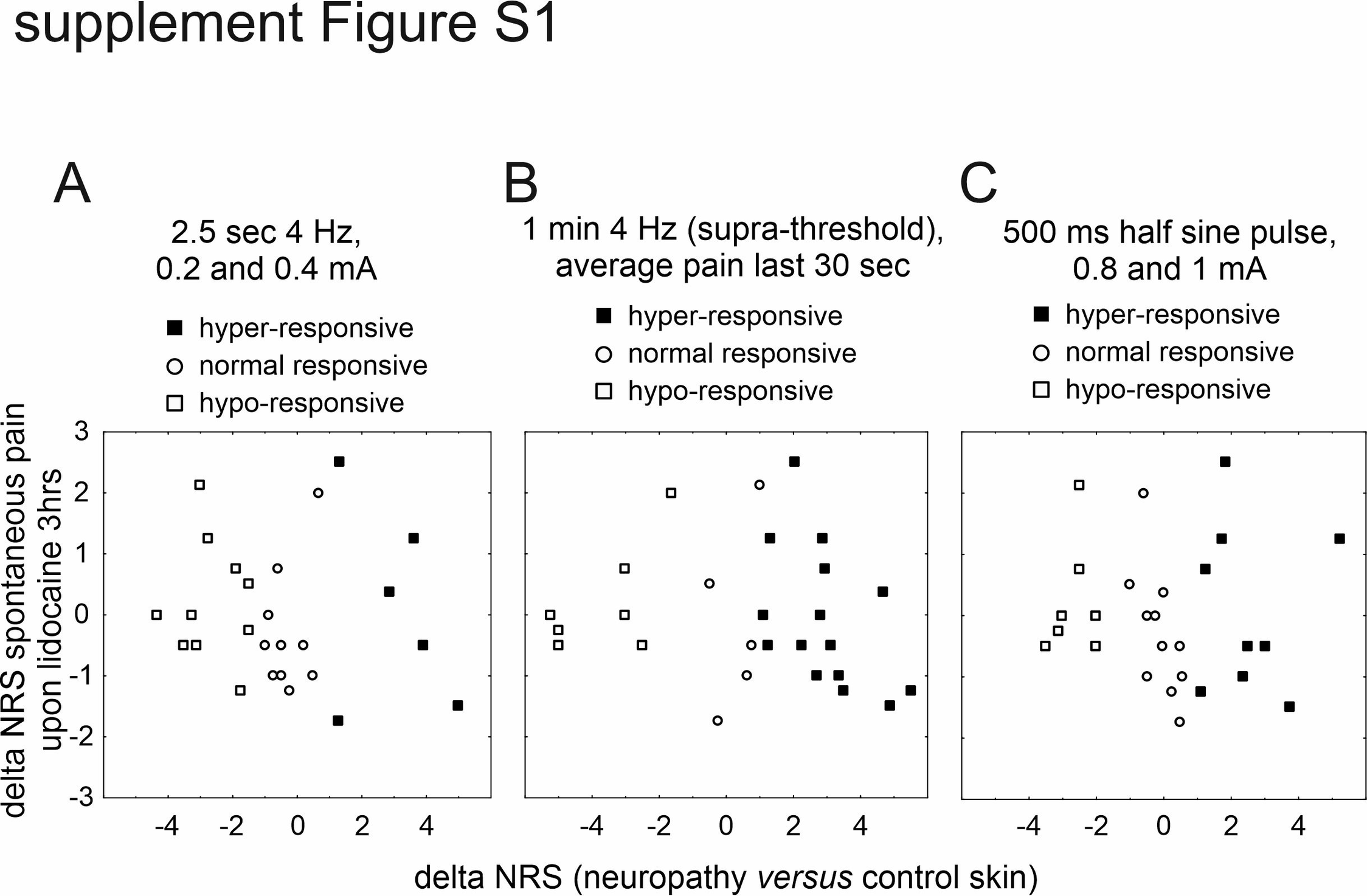

Supplement: S1 Fig — Analysis between pain reduction upon 3 hours lidocaine 5% patch (delta NRS, -10 to 10) and pain recorded from neuropathic (delta NRS from control skin sites) during (A) 2.5 sec 4 Hz sinusoidal pulses of 0.2 and 0.4 mA, (B) within the last 30 sec of 1 min 4 Hz supra-threshold sinusoidal pulses, and (C) upon 500 ms single half sine wave pulses of 0.8 and 1 mA. Patients are grouped “hyper-responsive” (solid squares”), “hypo-responsive” (open squares) or “normal responsive” (open circles) to transcutaneous electrical stimuli. (TIF) [file pone.0271327.s001.tif]

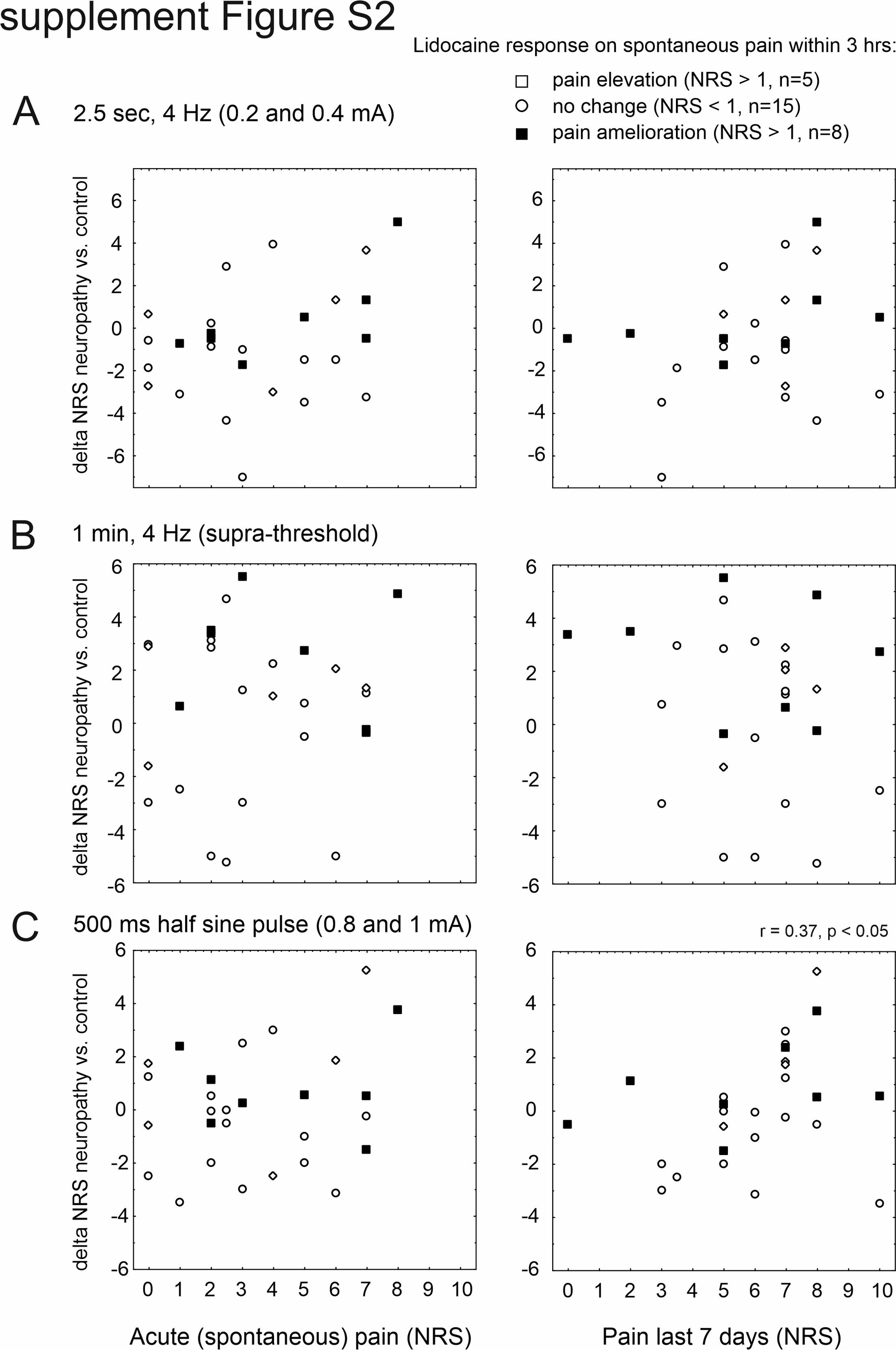

Supplement: S2 Fig — Analysis between pain from neuropathic skin sites (delta NRS neuropathy vs. control) and acute (spontaneous) pain (left panel) or maximum pain perceived during 7 days (right panel) upon (A) 2.5 sec 4 Hz sinusoidal pulses of 0.2 and 0.4 mA, (B) within the last 30 sec of 1 min 4 Hz supra-threshold sinusoidal pulses, and (C) upon 500 ms single half sine wave pulses of 0.8 and 1 mA. A significant correlation was identified between neuropathic skin site pain upon half sine wave stimulation and the 7-day maximum pain (p < 0.05). Scatterplot depicts patients’ groups by their lidocaine-patch response of acute (spontaneous) “pain-amelioration” (solid squares), “pain elevation” (open squares) or “no change” (open circles), respectively. (TIF) [file pone.0271327.s002.tif]
