# Supplementary material for: Local hyperexcitability of C-nociceptors may predict responsiveness to topical lidocaine in neuropathic pain
Source: PLoS One. 2022 Jul 14;17(7):e0271327. doi: 10.1371/journal.pone.0271327 (PMC9282664; doi:10.1371/journal.pone.0271327)
Supplement: Supplementary file 2 [file pone.0271327.s003.docx]

A)

Variables	Spearmans rank correlation significant correlations (p <.05)									
	co	pi	to	NR	acı	3hı	12h	NI	NF	NF
cold hyperal	1.	0.	-C	0.	-C	0.	0.	-C	-C	-C
pinprick hyp	0.	1.	0.	0.	-C	-C	0.	0.	-C	-C
allodynia to	-C	0.	1.	-C	**0.**	-C	**-C**	-C	0.	-C
NRS last 7 c	0.	0.	-C	1.	0.	0.	0.	0.	-C	**0.**
NRS acute (	-C	-C	**0.**	0.	1.	-C	-C	0.	0.	0.
NRS 3hrs lic	0.	-C	-C	0.	-C	1.	**0.**	-C	-C	-C
NRS 12hrs I	0.	0.	**-C**	0.	-C	**0.**	1.	-C	-C	-C
delta NRS 2	-C	0.	-C	0.	0.	-C	-C	1.	0.	**0.**
delta NRS 1	-C	-C	0.	-C	0.	-C	-C	0.	1.	0.
delta NRS 5	-C	-C	-C	**0.**	0.	-C	-C	**0.**	0.	1.

B)

Variable	Spearmans rank correlation significant correlations (p <.05) marked																			
	NI	NI	C	C	NF	NP	NF	CT	CTF	CT	NF	NF	Ct	Ct	NRS	acu	HAD	HA	SF	SF1
NP__2.5	1.	**0.**	0.	0.	**0.**	**0.**	**0.**	0.	0.	0.	**0.**	**0.**	0.	-0	0.	0.	0.	0.	-0	-0
NP__2.5	**0.**	1.	0.	**0.**	**0.**	**0.**	**0.**	**0.**	**0.**	0.	**0.**	**0.**	0.	0.	0.	0.	0.	0.	-0	-0
CTR__2.	0.	0.	1.	**0.**	0.	0.	0.	**0.**	**0.**	**0.**	0.	0.	**0.**	**0.**	-0	-0	0.	0.	-0	-0
CTR__2.	0.	**0.**	**0.**	1.	0.	0.	0.	**0.**	**0.**	**0.**	0.	**0.**	**0.**	**0.**	0.	-0	0.	0.	-0	-0
NP__1m	**0.**	**0.**	0.	0.	1.	**0.**	**0.**	**0.**	**0.**	0.	**0.**	**0.**	0.	0.	0.	0.	0.	0.	-0	0.
NP__1m	**0.**	**0.**	0.	0.	**0.**	1.	**0.**	**0.**	**0.**	0.	0.	0.	0.	-0	0.	0.	-0	0.	-0	0.
NP__1m	**0.**	**0.**	0.	0.	**0.**	**0.**	1.	**0.**	**0.**	0.	**0.**	**0.**	0.	0.	-0	-0	-0	0.	-0	0.
CTR__1r	0.	**0.**	**0.**	**0.**	**0.**	**0.**	**0.**	1.	**0.**	0.	**0.**	**0.**	**0.**	**0.**	-0	-0	**0.**	**0.**	-0	**-0**
CTR__1r	0.	**0.**	**0.**	**0.**	**0.**	**0.**	**0.**	**0.**	1.	**0.**	**0.**	**0.**	**0.**	**0.**	-0	-0	**0.**	**0.**	-0	**-0**
CTR__1r	0.	0.	**0.**	**0.**	0.	0.	0.	0.	**0.**	1.	0.	0.	0.	**0.**	0.	-0	-0	0.	0.	-0
NP__500	**0.**	**0.**	0.	0.	**0.**	0.	**0.**	**0.**	**0.**	0.	1.	**0.**	**0.**	**0.**	0.	-0	0.	0.	-0	-0
NP__500	**0.**	**0.**	0.	**0.**	**0.**	0.	**0.**	**0.**	**0.**	0.	**0.**	1.	**0.**	**0.**	0.	-0	0.	0.	-0	-0
Ctr__500	0.	0.	**0.**	**0.**	0.	0.	0.	**0.**	**0.**	0.	**0.**	**0.**	1.	**0.**	-0	-0	0.	0.	-0	**-0**
Ctr__500	-0	0.	**0.**	**0.**	0.	-0	0.	**0.**	**0.**	**0.**	**0.**	**0.**	**0.**	1.	-0	**-0**	0.	0.	-0	**-0**
NRS las	0.	0.	-0	0.	0.	0.	-0	-0	-0	0.	0.	0.	-0	-0	1.	0.	0.	-0	-0	-0
NRS ac	0.	0.	-0	-0	0.	0.	-0	-0	-0	-0	-0	-0	-0	**-0**	0.	1.	-0	-0	0.	-0
HADS__	0.	0.	0.	0.	0.	-0	-0	**0.**	**0.**	-0	0.	0.	0.	0.	0.	-0	1.	**0.**	**-0**	**-0**
HADS__	0.	0.	0.	0.	0.	0.	0.	**0.**	**0.**	0.	0.	0.	0.	0.	-0	-0	**0.**	1.	-0	**-0**
SF12__b	-0	-0	-0	-0	-0	-0	-0	-0	-0	0.	-0	-0	-0	-0	-0	0.	**-0**	-0	1.	0.
SF12__n	-0	-0	-0	-0	0.	0.	0.	**-0**	**-0**	-0	-0	-0	**-0**	**-0**	-0	-0	**-0**	**-0**	0.	1.
